# The Effect of GO Flake Size on Field-Effect Transistor (FET)-Based Biosensor Performance for Detection of Ions and PACAP 38

**DOI:** 10.3390/bios15020086

**Published:** 2025-02-05

**Authors:** Seungjun Lee, Jongdeok Park, Jaeyoon Song, Jae-Joon Lee, Jinsik Kim

**Affiliations:** 1 Department of Medical Biotechnology, College of Life Science and Biotechnology, Dongguk University, Seoul 04620, Republic of Korea; zldeja75@dgu.ac.kr (S.L.); thdwodbs95@dongguk.edu (J.S.); 2 Research Center for Photoenergy Harvesting & Conversion Technology (phct), Department of Energy and Materials Engineering, Dongguk University, Seoul 04620, Republic of Korea; whdejr213@dongguk.edu

**Keywords:** reduced graphene oxide (rGO), C/O ratio, surface area, rGO-FET, biomolecules

## Abstract

The performance development of rGO-FET biosensors by analyzing the influence of GO flake size on biosensing efficacy. GO flakes of varying sizes, from 1 µm to 20 µm, were prepared under controlled conditions, followed by characterization through SEM and XPS to evaluate their size, surface area, and C/O ratio. The biosensing performance was systematically assessed by rGO-FET biosensors, examining the effects of GO flake size, C/O ratio, and film thickness. PACAP38 was employed as a biomarker for receptor-mediated detection, while chlorine ions served as model analytes for receptor-free small molecule detection. The results indicate that decreasing the GO flake size enhanced the performance for both target biomolecules. These findings highlight the crucial importance of selecting GO flake sizes specific to target analytes and detection strategies, thereby optimizing biosensor efficiency.

## 1. Introduction

Graphene oxide (GO), developed as a cost-effective alternative to graphene, has garnered significant attention for its versatility in biosensing applications [1,2,3]. While pure graphene has exceptional electrical (1.04–1.05 × 10^2^ S/m) and thermal conductivity (5000 W·m^−1^·K^−1^) [4,5,6,7], its large-scale production remains prohibitively expensive, limiting its use in disposable biosensors. With its tunable electrical properties and cost-effective synthesis, GO offers sufficient conductivity for use as a semiconductor or insulator [8,9].

The unique properties of GO, including its surface area, functional groups, and electrical conductivity, are highly dependent on its synthesis method and degree of oxidation [10,11,12,13]. Synthesis methods such as Hummers’ method have enabled the production of high-quality GO with enhanced structural integrity and monolayer yields [14]. Further functionalization and reduction processes, such as chemical or thermal treatments, allow for tailoring GO into reduced graphene oxide (rGO), improving its electrical conductivity and performance for biosensor applications.

According to numerous studies, GO-based biosensors have wide applications and demonstrate high signal outputs [15,16,17,18,19,20,21,22,23,24,25,26,27,28,29,30,31]. Due to their high signal output and straightforward functionalization, GO-based sensors have recently been used to diagnose viral infections, including coronavirus [9,32,33,34]. Not only can electrical or electrochemical sensors be utilized, but GO also has applications in various optical sensing methods [35,36,37] and shows potential for wearable biosensor systems because of the ripples on its surface [32,38,39]. Many studies on GO-based biosensors have utilized receptors, including antibodies, aptamers, and enzymes, to enhance the selectivity of the devices, and the large number of functional groups in GO has facilitated this approach.

Since functional groups and electrical properties are essential in biosensor applications, controlling the size and functional groups of GO improves its sensitivity and performance. GO’s versatility as a biosensor continuously expands. Controlling GO flakes’ size and surface chemistry offers a promising route to optimizing biosensor performance [40,41,42]. Composite materials incorporating GO with other nanostructures expand its potential in device functionalities [31,35,41,42,43,44,45,46,47,48,49].

**Figure 1:**
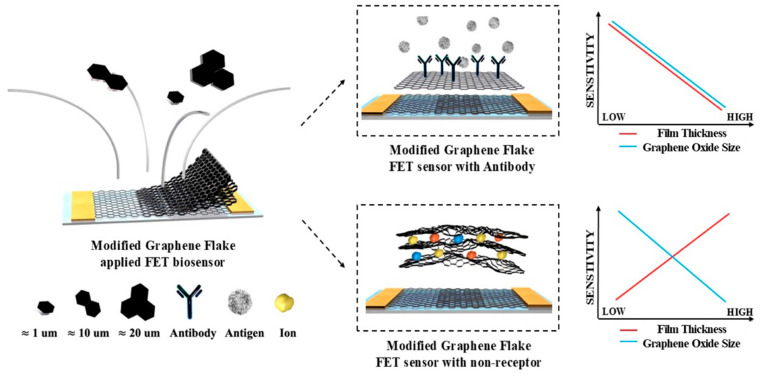
Schematic showing the utilization of ≈1, ≈10, and ≈20 μm size of graphene flakes to verify rGO-FET biosensor sensitivity differences by controlling the graphene flake size.

In this study, we comprehensively investigate the role of GO flake size in biosensing performance. GO flakes of varying sizes (1–20 µm) were synthesized, and their structural and chemical properties were analyzed using SEM and XPS. By employing rGO-FET biosensors fabricated through MDD methods, we evaluated the effects of GO size, film thickness, and C/O ratio on sensor sensitivity. Biomarker detection experiments using PACAP 38 and chlorine ions revealed that smaller GO flakes exhibited superior performance due to their higher surface area and optimized functional group density as shown in Figure 1. These findings underscore the importance of tailoring the GO flake size to the target biomolecule and sensing application, offering new insights into the development of high-performance biosensors.

## 2. Materials and Methods

### 2.1. Graphene Oxide Synthesis and Purification

GO was synthesized using a modified Hummer’s method and prepared with sizes of ≈1, ≈10, and ≈20 μm [50]. The ≈1 μm size GO utilized commercial GO (Graphene Supermarket, NY, USA), while the ≈10 and ≈20 μm sizes were synthesized using the following methods. Graphite powder was prepared using an electrochemical exfoliation method [51]. An amount of 0.8 g of graphite powder, 0.8 g of NaNO_3_, and 37 mL of H_2_SO_4_ (95.0–97.0%, Sigma Aldrich, St. Louis, MO, USA) was added to a round flask in an ice bath environment. Then, 3.2 g KMnO_4_ was slowly added to a round flask under stirring. The round flask was then fixed to a heating mantle. The mixed solution was heated to 35 ± 5 °C for 60 min with stirring. Then, 64 mL of deionized water (DIW) was added to the mixture, which was stirred for 30 min while increasing the temperature to 90 ± 5 °C. After removing the heat source, 160 mL of DIW was added to the solution. Lastly, 4.8 mL of H_2_O_2_ (30.0% (*w*/*w*) in H_2_O, Sigma-Aldrich) was added.

The synthesized GO solution mixture was filtered and washed with 100 mL of 3.5–3.7% HCl and a large amount of DIW using a vacuum filtration system. Subsequently, the GO slurry was dispersed in DIW using a mechanical shaker. The supernatant was collected by centrifuging it at 1000 rpm for 2 min to remove unreacted graphite. The collected supernatant was centrifuged at 4000 rpm for 60 min to collect the precipitate. The precipitate was redispersed in a 6:1 volume ratio of ethyl alcohol and DIW by sonication at 120 W for 500 s. The dispersion solution was centrifuged at 4000 rpm for 80 min to collect the supernatant and precipitate. Then, the supernatant was re-exfoliated by sonicating it at 120 W for 600 s, and the solution was marked as ≈10 μm size flakes. The precipitate was redispersed in a 6:1 volume ratio of ethyl alcohol to DIW by mechanical shaking. The dispersion solution was centrifuged at 4000 rpm for 35 min to collect the supernatant, which was marked as ≈20 μm size flake. A schematic of GO flake synthesis and purification is shown in Figure S1.

### 2.2. Graphene Oxide Flake Characterization

Scanning electron microscopy (SEM) measurements were carried out using a Sigma 300 VP FE-SEM (Carl Zeiss AG, Oberkochen, Germany) to analyze the size of the GO sheets and the surface morphology of the Langmuir–Blodgett (LB) films. SEM images were obtained at a low acceleration voltage of 1 kV in SE2 and lens modes.

X-Ray Photoelectron Spectroscopy (XPS, Versaprobe II, ULVAC-PHI, Chigasaki, Japan) was performed to analyze the atomic components and bonding structures of the GO and rGO films. Spectra were obtained at 15 kV using a monochromatic Al-Kα excitation source.

### 2.3. Graphene Oxide-Based Biosensor Fabrication

The rGO-FET biosensors were fabricated on silicon dioxide (SiO_2_) wafers, following methods in the previous research [52]. Six rectangular patterns (50 μm × 100 μm each) in a vertical parallel line covering patterned GO on both sides with gold electrodes were produced by meniscus-dragging deposition (MDD) for GO deposition, the reduction of GO, photolithography, reactive ion etching (RIE), e-beam evaporation, and the lift-off process. After applying O_2_ plasma (CUTE, Femto Science, Gyeonggi-do, Republic of Korea) at 50 W and 50 sccm for 50 s, a 1:19 ratio of 3-aminopropyl triethoxysilane (APTES, Merck, Darmstadt, Germany) and 99% ethyl alcohol (Merck, Darmstadt, Germany) were used to treat the surface of the SiO2 wafer to create appropriate conditions for the deposition of GO. The MDD method was used to deposit GO on the treated SiO_2_ wafers. After the deposition of GO, 57wt% of hydriodic acid (Sigma-Aldrich Inc.) was used for 2 h 30 min at 80 °C for thermal reduction to obtain electrical conductivity. Photolithography, Mask Aligner (MDA-400S, Midas System, Daejeon, Republic of Korea) was utilized for the photoresist (GXR 601, Merck KGaA, Darmstadt, Germany), deposited by spin coating on a reduced stock/flake GO wafer to obtain the proper pattern. AZ 300 MIF (Merck KGaA, Darmstadt, Germany) was used to develop the solution, and the light-absorbed area on the wafer was removed. The PR on the pattern and the remaining rGO, other than the pattern, were removed by RIE using oxygen gas. PR coating and photolithography were processed to perform electrode patterning. Development was applied to remove the PR from the electrode patterned area. Then, the Au source was evaporated in 2000 Å in 2 Å/s, to deposit itself by e-beam evaporator (ULVAC, MA, USA) onto the entire wafer. Finally, an acetone wash was performed to remove the Au deposited on the wafer beside the electrode region. The photograph and detailed dimensions of the fabricated sensor are shown in Figure S2.

### 2.4. Biomarker and Ion Detection

Pituitary adenylate cyclase-activating polypeptide-38 (PACAP38) and chlorine ions were utilized as biomarkers and ions to demonstrate sensor performance.

In PACAP 38 detection, the PACAP/ADCYAP1 antibody (Novus Biologicals LLC, CO, USA) was immobilized using ethyl-(dimethylaminopropyl) carbodiimide (EDC) and N-hydroxysuccinimide (NHS) (both from Sigma-Aldrich Inc.) in a 1XPBS (Gibco, NY, USA) solution. A mixture of 2 mM EDC and 8 mM NHS, along with 10 µg/mL of antibody, reacted with the sensor for 90 min. After that, the PACAP 38 antigen was diluted with 1XPBS and reacted with the sensor for 30 min.

For chlorine ion detection, chlorine water (Kanto Chemical, Tokyo, Japan) was diluted with DIW to obtain an appropriate concentration and allowed to react for 30 min.

To detect the biomarker PACAP 38 and the chlorine ion, I–V characterization was conducted over the range of 0 to 5 V. The average resistance within this range was calculated, and the resulting resistance values were compared before and after the reaction with the biomarker and ion to assess the sensor performance.

## 3. Results and Discussion

### 3.1. Surface Area Analysis of Size-Controlled GO Flake

**Figure 2:**
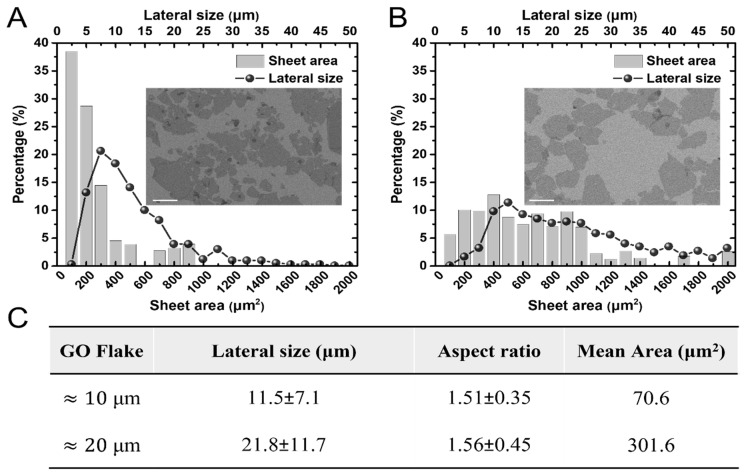
Size analysis of as-prepared GO flakes. (**A**,**B**) show the size and area distributions of the ≈10 and ≈20 μm GO flakes, respectively. The scale bar of each SEM image is 10 μm. (**C**) Morphological characteristics of prepared GO flakes. Variation values of the major axis, minor axis, area, and aspect ratio of the GO flakes.

The effect of the GO sheet size on the electrochemical sensitivity was investigated. GO was prepared as two types of solutions, with the sheet size controlled by adjusting the exfoliation and centrifugation steps. Subsequently, each solution was transferred to a pretreated silicon wafer using the LB technique. The size distribution of each individual sheet on the silicon wafer of the as-prepared GO LB film was investigated using SEM. The lateral length size was confirmed by examining 380 and 442 samples for the ≈20 and ≈10 μm solutions, respectively. Furthermore, the average mean area of the sheets was calculated by examining 315 and 317 samples of ≈20 and ≈10 μm, respectively, using ImageJ software (v. 1.54g, National Institutes of Health, MD, USA). As shown in Figure 2, the average lateral size major length and area were 21.8 ± 11.7 μm (301.6 μm^2^) and 11.5 ± 7.1 μm (70.6 μm^2^), respectively.

### 3.2. Functional Group Analysis of Size-Controlled GO Flakes

XPS analysis was performed to investigate the oxygen composition of the as-prepared GO film and the chemically reduced GO films. The GO film was deposited on a pretreated silicon wafer using the MDD method. for XPS analysis. Subsequently, the GO film was reduced using the chemical method. In particular, the components of the O1s spectra were significantly reduced, indicating that the GO film was successfully reduced. As shown in Figure S3, the peaks of C1s and O1s for carbon and oxygen were confirmed at ca 284.5 eV and 531.0 eV. At the high resolution of the C1s and O1s spectra of the GO and rGO films, peaks were observed at ca. 284.1~289.3 eV and ca. 530.1~549.9 eV, which can be described as bonding structures such as C=C, C-C, C-O, C=O, COOH, C=O, C-O-C, C-O, and H_2_O. These results were consistent with those reported in the literature [53,54,55]. Furthermore, the C/O ratio was analyzed to compare the oxygen content of each sample. As shown in Table 1, the C/O ratios of the GO films composed at different sizes of ≈1, ≈10, and ≈20 μm were 1.31, 2.18, and 2.75, respectively. However, the chemically reduced GO film had a significantly decreased oxygen content and showed C/O ratios of 2.79, 7.20, and 7.77, depending on the size. The oxygen content tended to decrease as the flake size increased, because the majority of the oxygen functional groups were located at the edges. Therefore, the ≈1 μm rGO film showed the highest oxygen content of 26.4%, which was higher than the ≈10 and ≈20 μm rGO films.

### 3.3. Detection Characteristic of Biomolecule Depending on GO Flake Size

Depending on the size of the GO flakes, different amounts of flakes could be deposited in the same area. Because functional groups exist at the edge of the GO flake, the number of functional groups is different based on the same amount of GO flakes deposited on an equal area.

**Figure 3:**
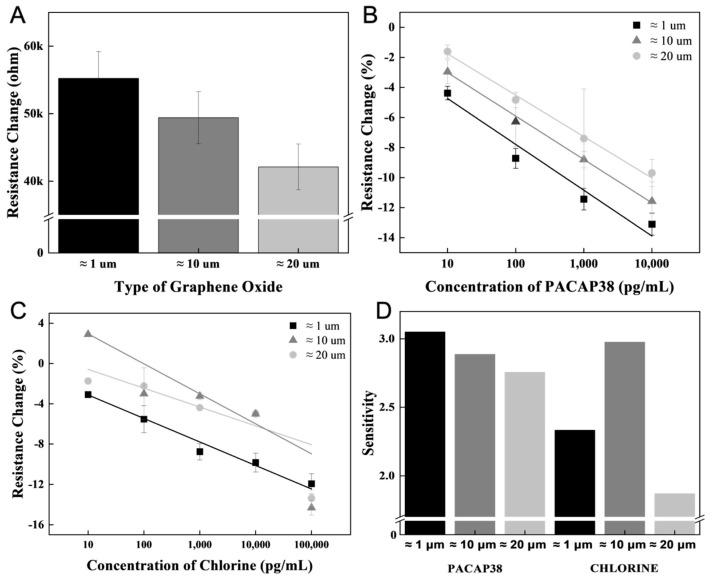
PACAP38 and chlorine detection using a controlled GO flake. (**A**) Resistance changes according to receptor immobilization depending on the GO flake size. (**B**) PACAP38 detection in the concentration range of 10 pg/mL~10 ng/mL for GO flakes of different sizes. (**C**) Linear slope of chlorine detection within a certain concentration range. (**D**) PACAP38 and chlorine detection slope of the linear function analysis with ≈1, ≈10, and ≈20 μm size of GO flakes.

Accordingly, the receptor immobilization rate could be increased by adjusting the size of the GO flakes. A high resistance was measured after receptor immobilization on a small flake size of ≈1 μm (Figure 3A). To analyze the effect of GO flake size on biomarker measurement, 10 pg/mL to 10 ng/mL concentrations of biomarkers were measured in the receptor-immobilized sensor (Figure 3B). Depending on the size of the GO flake, when the same amount is applied to the same area, the number of small-sized biomaterials, such as ions, permeates differently between the GO flake sheets. When the GO flake size was large, the ion measurement efficiency was higher than that of the small GO flakes (Figure 3C). The difference in the slope of the linear function of the measured result according to the concentration was verified, and the smaller the GO flake size, the higher the measurement efficiency of biomarkers such as PACAP38 measured with a receptor (Figure 3D).

**Figure 4:**
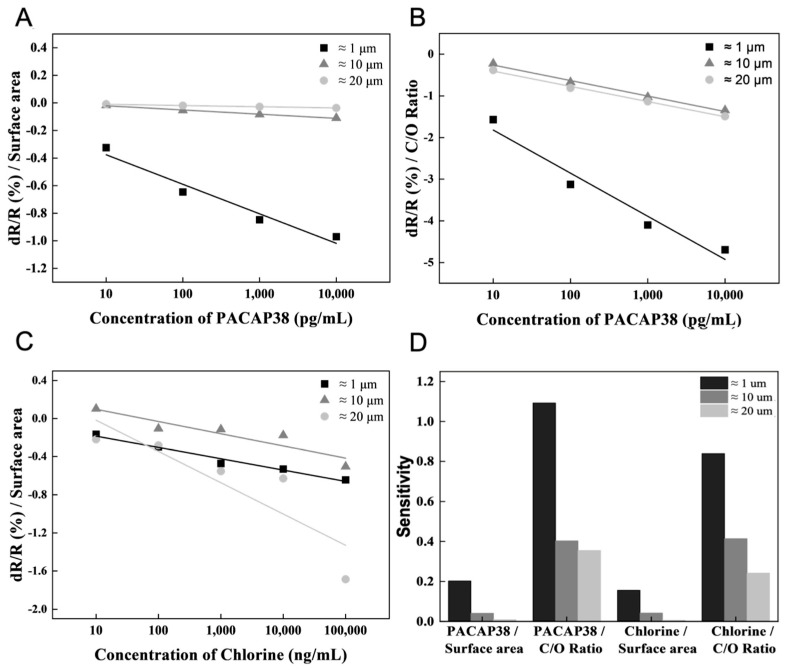
PACAP38 and chlorine detection analysis by GO flake surface area and C/O ratio. (**A**) and (**B**) Normalized PACAP38 detection results by surface area and C/O ratio of GO flakes, respectively. (**C**) Normalized chlorine detection results based on the GO flake surface area. (**D**) The sensitivity from the normalized result of each GO flake.

The size-controlled GO flakes were used to detect PACAP38 and chlorine. To analyze the size and functional groups of the GO flakes, the surface area was calculated from the major and minor axis distances of the GO flakes, and the C/O ratio of the GO flakes was determined using XPS analysis. The ≈1, ≈10 and ≈20 μm size of GO flake surface areas were calculated as 2.5 μm^2^, 88.16 μm^2^, and 315.36 μm^2^, respectively. Based on the ≈1, ≈10 and ≈20 μm GO flakes, the C/O ratio was obtained by XPS analysis, and the PACAP38 detection results were normalized. The result value (dR/R (%)) measured for each concentration of PACAP38 was divided by each C/O ratio for normalization. The normalized value is shown as a graph, and the difference in the slope value was confirmed by linearly fitting the normalized value according to the GO flake surface area and C/O ratio in PACAP38 detection (Figure 4A,B). The chlorine detection results were normalized based on the calculated ≈1, ≈10, and ≈20 μm GO flake surface areas. The slope values were derived via linear fitting by plotting the normalized values for each concentration (Figure 4C).

It can be argued that PACAP38 and chlorine have different effects when measuring PACAP38 or chlorine, as the detection methods for PACAP38 and chlorine are different, namely normalization based on surface area or C/O ratio; the large difference in the linear fit slope value is derived accordingly. In PACAP38 detection, a greater effect is derived from the C/O ratio, such as the functional group, rather than the surface of the graphene flake. A ≈1 μm size graphene flake has more functional groups that could be exposed to the surface in the same area. The small size of the substance requiring a receptor, such as PACAP38, is advantageous for detection. However, for chlorine ions, which can be detected without a receptor, the sensitivity exhibited a more complex correlation with the graphene flake size and functional group ratio. In the case of chlorine, because detection is achieved without needing a receptor, smaller graphene flakes with a higher density of functional groups provide more opportunities for ions to penetrate the inter-flake spaces, thereby resulting in higher sensitivity (Figure 4D).

### 3.4. Detection Characteristics of a Biomolecule with Deposition-Controlled GO Flake

**Figure 5:**
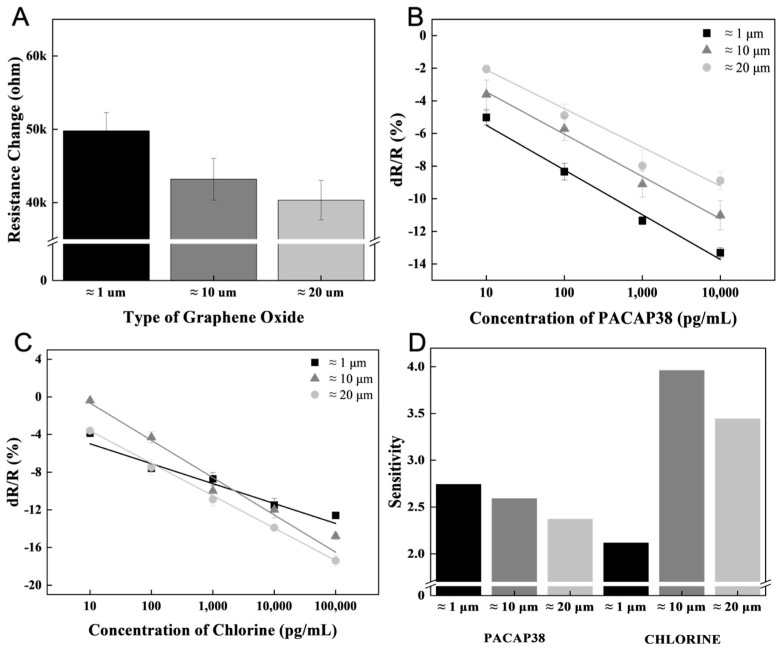
PACAP38 and chlorine detection with controlled GO flake 30 depositions. (**A**) Resistance changes according to receptor immobilization depending on GO flake size. (**B**) PACAP38 detection in the concentration range of 10~100 ng/mL in GO flakes of different sizes. (**C**) The linear function slope of chlorine detection in a certain range of concentrations. (**D**) PACAP38 and chlorine detection slope of linear function analysis with ≈1, ≈10, and ≈20 μm size of GO flakes.

The highest resistance increased after receptor immobilization at ≈1 μm, and immobilization decreased as the flake size increased at ≈1, ≈10, and ≈20 μm (Figure 5A). A concentration of 10~10,000 pg/mL of PACAP38 was detected with three different sizes of graphene flakes (Figure 5B). Quantitative detection was secured under all flake size conditions by linearization. In addition, a 10~100,000 pg/mL concentration of chlorine was measured under different graphene flake size conditions (Figure 5C). To analyze the sensitivity, the slope of the linear function of the measured results is shown (Figure 5D).

In addition to the biosensor fabricated with 20 depositions, a rGO-FET biosensor with 30 depositions was fabricated, and PACAP38 was measured in the same manner. Consistent with the previous description, PACAP38 can be detected where a specific receptor is immobilized. PACAP38 detection depends on the functional groups of the graphene. Therefore, even if the conditions of the biosensor were changed by applying more graphene, significant changes in the measured values and sensitivities were not observed.

**Figure 6:**
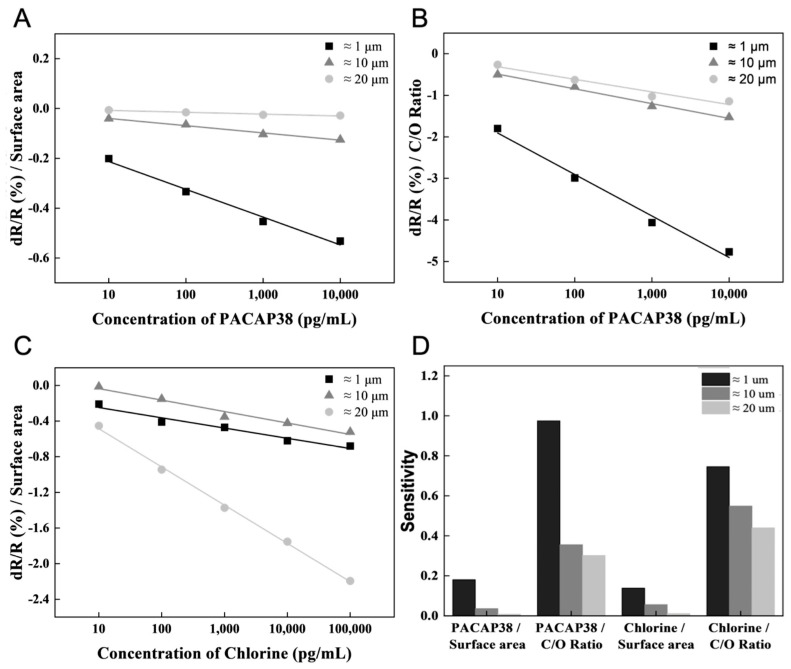
Thirty depositions condition of the graphene biosensor for PACAP38 and chlorine detection analysis based on GO flake surface area and C/O ratio. (**A**,**B**) Normalized PACAP38 detection result by surface area and C/O ratio of GO flake, respectively. (**C**) Normalized chlorine detection results for GO flake surface area. (**D**) The sensitivity from the normalized result of each GO flake.

To analyze the sensitivity according to the graphene flake size, the results of the biosensor deposited 30 times were analyzed in the same manner as shown in Figure 4. The analysis of the PACAP38 detection results by surface area and C/O ratio is shown in Figure 6A,B. The chlorine detection analysis was based on the surface area (Figure 6C). In addition, the biosensor sensitivity according to flake size and deposition conditions was analyzed by comparing the slopes of the graphs (Figure 6D).

The analysis according to the PACAP38 measurement under the 30 depositions condition did not differ significantly from the sensitivity derived under the 20 depositions condition. The graphene functional group exposed to the surface in the same area was not affected by the thickness control. However, chlorine, measured by penetrating the graphene layer, can improve the sensitivity of biosensors with thick graphene layers. Similarly, for chlorine ions, which are detected by penetrating the graphene layer, results consistent with the 20 depositions condition were obtained. The C/O ratio remained largely unaffected by varying the GO deposition conditions, and the sensitivity for chlorine detection was the highest when the C/O ratio was elevated in larger graphene flakes. This can be attributed to the fact that without receptor immobilization, smaller graphene flakes with a higher density of functional groups capture more chlorine ions within the graphene network, leading to enhanced detection sensitivity (Figure 6D). Furthermore, the C/O ratio was not affected by chlorine detection with different GO depositions, and there was no significant slope value difference compared with the 20 depositions condition analysis of the sensor (Figure S4).

### 3.5. Comparative Analysis According to Graphene Deposition Conditions

**Figure 7:**
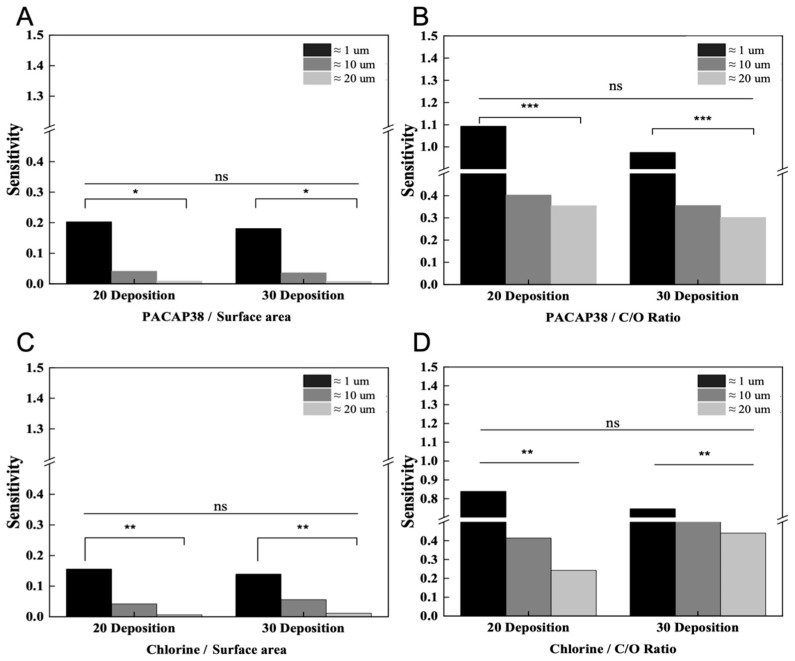
Analysis of the effect of the graphene C/O ratio or surface area through sensitivity derived from the measured values of PACAP38 and chlorine measured in 20 and 30 depositions condition sensors implemented using graphene flakes of sizes ≈1, ≈10, and ≈20 μm. The *p*-values based on the number of depositions and flake sizes were determined using *t*-tests and are represented as follows: *** *p* ≤ 0.001, ** *p* ≤ 0.01, * *p* ≤ 0.05, and ns (not significant) for *p* > 0.05. (**A**,**B**) Analysis of the sensitivity values obtained by measuring PACAP38 in 20 and 30 deposition sensors as surface area and C/O ratio. (**C**,**D**) Sensitivity values obtained by measuring chlorine as surface area and C/O ratio analyzed by value.

To analyze the fact that small substances such as ions can be measured without a receptor in a sensor implemented by layering graphene flakes and to analyze the difference from the measurement of biomaterials using existing receptors, we used a sensor with layers of different thicknesses and graphene flakes. The measured values are shown in Figure 7, which indicates that the significant difference between the 20 and 30 deposition groups is insignificant and * indicates the significance of the sensitivity change value according to the graphene flake size.

The receptors are bound to the graphene functional group, and the reaction occurs on the surface of graphene in such a way that the target biomaterial can be measured. Therefore, as shown in Figure 7A,B, it can be confirmed that there is no difference despite the difference in graphene deposition. This proves that the response of the graphene surface has a low correlation with the thickness according to the deposition. Furthermore, small-sized ions such as chloride penetrate the graphene layers, thereby altering the electrical conductivity of the FET sensor. As illustrated in Figure 7C,D, it is evident that smaller graphene flakes facilitate the formation of a network within the graphene layer, providing ample space for penetration. This suggests that chloride ions can be detected more effectively. This interpretation can be understood by analyzing the results obtained without the use of chloride detection receptors.

## 4. Conclusions

With adjustments to the regulation of the graphene flake size, we examined the originality of the graphene-based FET biosensors. XPS was used to determine the ratio of carbon to oxygen in the graphene oxide flakes. Electrical conductivity can vary depending on the thickness and frequency of graphene flake application; the constructed sensor has a modest impact on conductivity differences.

In measuring specific PACAP38 using the existing immunoassay method, the antibody (receptor)–antigen method, performance can be increased depending on the extent to which the functional groups of GO are exposed. To determine the effect of functional groups that can be included in the same area according to the size of the graphene flakes, PACAP38 and ions (non-receptor immobilization on graphene flakes) were detected using a sensor made of graphene flakes of different sizes. By analyzing the synthesized graphene flakes of different sizes using XPS, the carbon and oxygen content ratio was analyzed, and the sensor performance was confirmed using the biomaterial detection value of the produced graphene flake-based sensor. In addition, using the surface area according to the graphene flake size, the sensor performance according to the detection value of the biomaterial was analyzed and compared with the C/O ratio.

Target substances requiring receptors, such as PACAP38, exhibit improved performance as the size of the graphene flakes decreases. Conversely, for substances such as ions that can be measured without receptors, variations in the ratio of functional groups due to changes in the graphene flake size allow us to elucidate the principles of ion detection and the ease of biomaterial detection.

In conclusion, the role of the graphene functional groups is crucial for the detection of biomaterials. Direct detection methods, such as ion measurements and graphene flake size control, are essential for effective sensing. Thus, by optimizing the ratio of the functional groups according to the size of the graphene flakes, it is possible to implement a robust biosensor tailored to the target biomaterial.

## Figures and Tables

**Table 1:** Graphene C/O ratio analysis by XPS for different sizes of GO and chemically reduced GO.

Atomic Percentage (%)	≈1 μm		≈10 μm		≈20 μm	
	GO	rGO	GO	rGO	GO	rGO
Carbon	56.7	73.6	68.6	87.8	73.3	88.6
Oxygen	43.3	26.4	31.4	12.2	26.7	11.4
C/O ratio	1.31	2.79	2.18	7.20	2.75	7.77

## Data Availability

The data presented in this study are available from the corresponding authors upon request.

## Supplementary Materials

The following supporting information can be downloaded at: https://www.mdpi.com/article/10.3390/bios15020086/s1, Figure S1: Graphene flake synthesis and purification; Figure S2: Photograph of fabricated rGO-FET biosensor and dimension of the substrate, device, and the rGO channel; Figure S3: High-resolution XPS C1s and O1s spectra of ≈1 μm (A, D), ≈10 μm (B, E) and ≈20 μm (C, F) corresponding to the as-prepared graphene oxide and chemically reduced graphene oxide films, respectively; Figure S4: Analysis of Chlorine Detection Sensitivity in Biosensors Implemented with Graphene Flakes of Different Sizes: Normalized Sensor Sensitivity According to the C/O Ratio Based on Graphene Size. References [55,56,57,58] are cited in the Supplementary Materials.
