# Supplementary material for: The Effect of GO Flake Size on Field-Effect Transistor (FET)-Based Biosensor Performance for Detection of Ions and PACAP 38
Source: Biosensors (Basel). 2025 Feb 5;15(2):86. doi: 10.3390/bios15020086 (PMC11853402; doi:10.3390/bios15020086)
Supplement: Supplementary file 1 [file biosensors-15-00086-s001.zip › biosensors-3427211-supplementary.pdf]

Supplementary Information

The Effect of GO Flake Size on Field Effect Transistor (FET) based Biosensors Performance for Detection of Ion and PACAP 38

Seungjun Lee[1, †], Jongdeok Park [b,1], Jaeyoon Song[a], Jae-Joon Lee [b*], Jinsik Kim[a*]

[1] Department of Medical Biotechnology, College of Life Science and Biotechnology,

Dongguk University, Seoul 04620, Republic of Korea

[2] Department of Energy & Materials Engineering, Dongguk University, Seoul 04620,

Republic of Korea

*Correspondence: jjlee@dongguk.edu, Tel: +82-2-2260-4979 (J.-J.L.); lookup2@dongguk.edu, Tel.: +82-31-961-5155 (J.K.);

† These authors contributed equally to this work.

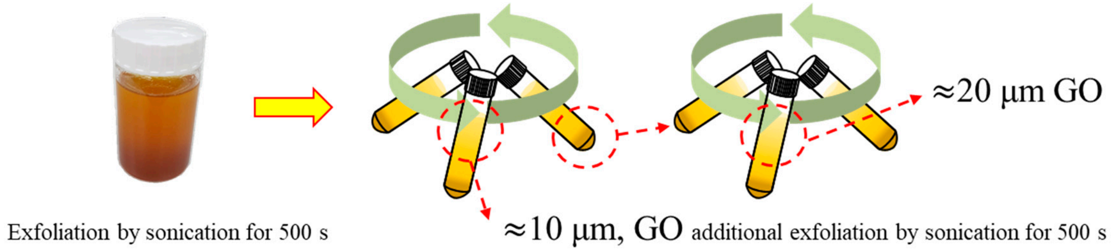

Figure S1. Graphene flake synthesis and purification.

Graphite powder dissolved sample was sonicated and exfoliated at middle power (120 w) for 500 s. Then, dispersed graphene flake was purified by specific condition of centrifuge such as 4000 rpm for 80 min. Approximately 10 um (11.6 um) of graphene flake was given by additional sonication treatment at 120 w for 600 s. Furthermore, 21.9 um (appx. 20 um) graphene flake was confirmed further centrifuge applying. Thus, two different size of graphene flake was synthesized by particular condition of sonication and centrifuge optimization.

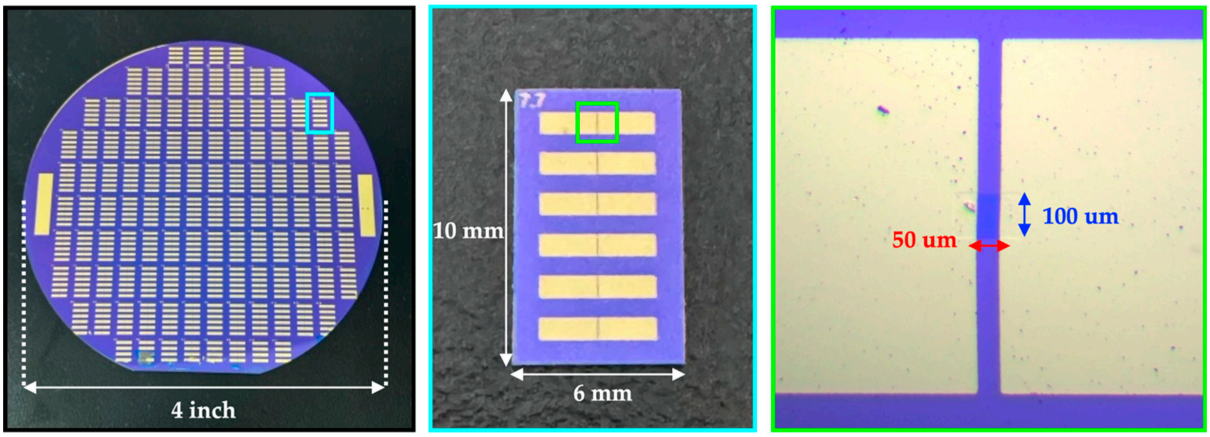

Figure S2. Photograph of fabricated rGO-FET biosensor and dimension of the substrate, device, and the rGO channel.

As shown in Figure S2, the rGO-FET biosensor was fabricated on a 4-inch SiO_2 wafer using the MDD method and photolithography process. Six sensors were combined into a single device, measuring 10 mm by 6 mm. The sensor was designed as a 2-terminal device and features an rGO channel with a length of 50 μm and a width of 100μm.

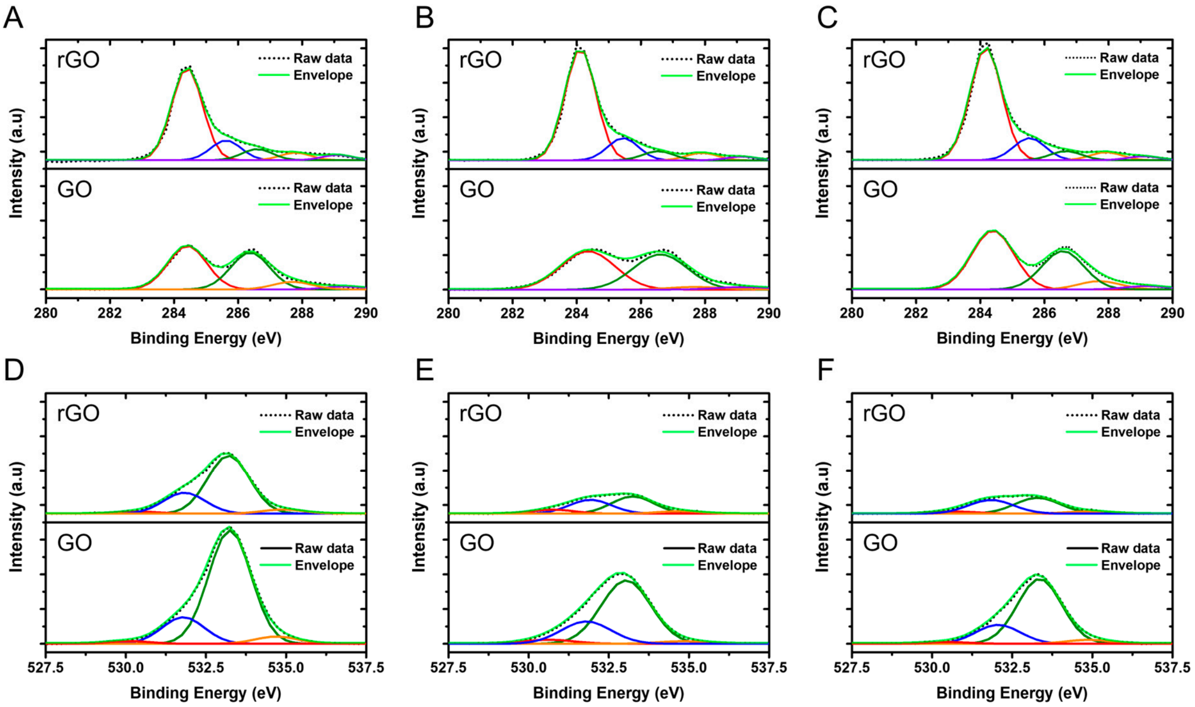

Figure S3. High-resolution XPS C1s and O1s spectra of ≈ 1 μm (A, D), ≈ 10 μm (B, E) and ≈ 20 μm (C, F) corresponding to the as-prepared graphene oxide and chemically reduced graphene oxide films, respectively.

Figure S3 shows the C1s and O1s XPS spectra of as-prepared graphene oxide and chemically reduced graphene oxide films with different lateral size (≈1 μm, ≈10 μm, ≈20 μm). In the high-resolution of C1s XPS spectra, distinct peaks corresponding to ca. 284.5 eV and ca. 286.5 eV related to sp2 C=C and C-O bonds, were observed. Subsequently, the peak at ca. 284.5 eV dramatically increased due to the restoration of the structures by the chemical reduction. Furthermore, the intensity of the O1s spectra generally decreased owing to the removal of the oxygen functionalities such as C=O, O-H, C-O-C, C-O, and H_2O in chemically reduced graphene oxide. Therefore, these results were consistent with previous reports. [55–58] The ≈ 1 μm GO film exhibited higher oxygen contents than ≈ 10 μm and ≈ 20 μm GO films both before and after reduction. This trend is consistent with the C/O ratios reported in

the results (Table 1).

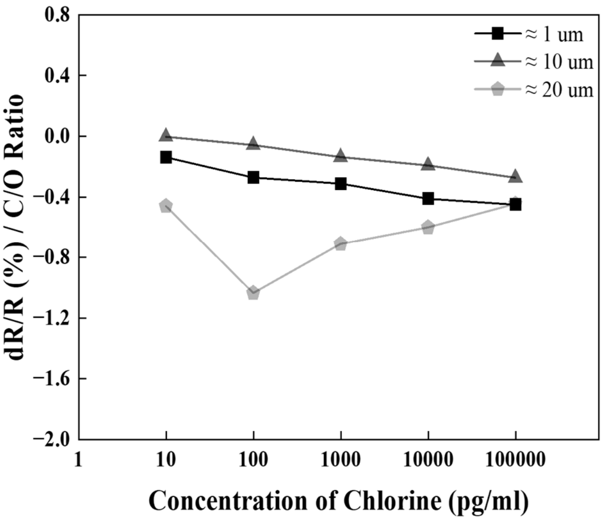

Figure S4. Analysis of Chlorine Detection Sensitivity in Biosensors Implemented with Graphene Flakes of Different Sizes: Normalized Sensor Sensitivity According to the C/O Ratio Based on Graphene Size.

In this study, chlorine detection at concentrations ranging from 10 pg/ml to 100 ng/ml was performed using graphene oxide (GO) flakes of ca. 1 μm, 10 μm, and 20 μm to analyze the influence of functional groups on the GO flakes. After normalizing the results based on the C/O ratio, it was observed that the slopes, representing the sensitivity for each flake size, showed minimal differences. Therefore, for detecting small ions, such as chlorine, the impact of the functional groups present in graphene is negligible.
